# Preventing adverse events of chemotherapy for gastrointestinal cancer by educating patients about the nocebo effect: a randomized-controlled trial

**DOI:** 10.1186/s12885-022-10089-2

**Published:** 2022-09-23

**Authors:** T. Michnevich, Y. Pan, A. Hendi, K. Oechsle, A. Stein, Y. Nestoriuc

**Affiliations:** 1 Department of Psychosomatic Medicine and Psychotherapy, University Medical Center Hamburg-Eppendorf, Hamburg, Germany; 2 Department of Psychiatry and Psychotherapy, Charité – University Medicine Berlin, Berlin, Germany; 3 Present address: Charité – University Medicine Berlin, Berlin, Germany; 4 Department of Oncology, Asklepios Clinic Barmbek, Hamburg, Germany; 5 Center of Oncology, University Medical Center Hamburg-Eppendorf, Hamburg, Germany; 6 Department of Clinical Psychology, Helmut Schmidt University, University of the Federal Armed Forces Hamburg, Hamburg, Germany; 7 Department of Systems Neuroscience, University Medical Center Hamburg-Eppendorf, Hamburg, Germany

**Keywords:** Placebo, Nocebo, Expectations, Chemotherapy, Adverse events

## Abstract

**Background:**

Adverse events of chemotherapy may be caused by pharmacodynamics or psychological factors such as negative expectations, which constitute nocebo effects. In a randomized controlled trial, we examined whether educating patients about the nocebo effect is efficacious in reducing the intensity of self-reported adverse events.

**Methods:**

In this proof-of-concept study, *N* = 100 outpatients (mean age: 60.2 years, 65% male, 54% UICC tumour stage IV) starting first-line, de novo chemotherapy for gastrointestinal cancers were randomized 1:1 to a nocebo education (*n* = 49) or an attention control group (*n* = 51). Our primary outcome was patient-rated intensity of four chemotherapy-specific and three non-specific adverse events (rated on 11-point Likert scales) at 10-days and 12-weeks after the first course of chemotherapy. Secondary outcomes included perceived control of adverse events and tendency to misattribute symptoms.

**Results:**

General linear models indicated that intensity of adverse events differed at 12-weeks after the first course of chemotherapy (mean difference: 4.04, 95% CI [0.72, 7.36], *p* = .02, *d* = 0.48), with lower levels in the nocebo education group. This was attributable to less non-specific adverse events (mean difference: 0.39, 95% CI [0.04, 0.73], *p* = .03, *d* = 0.44) and a trend towards less specific adverse events in the nocebo education group (mean difference: 0.36, 95% CI [− 0.02, 0.74], *p* = .07, *d* = 0.37). We found no difference in adverse events at 10-days follow-up, perceived control of adverse events, or tendency to misattribute non-specific symptoms to the chemotherapy.

**Conclusions:**

This study provides first proof-of-concept evidence for the efficacy of a brief information session in preventing adverse events of chemotherapy. However, results regarding patient-reported outcomes cannot rule out response biases. Informing patients about the nocebo effect may be an innovative and clinically feasible intervention for reducing the burden of adverse events.

**Trial registration:**

Retrospectively registered on March 27, 2018 to the German Clinical Trial Register (ID: DRKS00009501).

**Supplementary Information:**

The online version contains supplementary material available at 10.1186/s12885-022-10089-2.

## Background

The overwhelming majority of patients undergoing chemotherapy for gastrointestinal (GI) cancer is affected by treatment-related adverse events (AEs) [1, 2]. Among the most reported by patients are fatigue (88%), diarrhoea (75%), constipation (73%) and vomiting (58%) [1].

Aside from impairing patients’ quality of life (QoL) [3, 4], AEs are associated with decreased treatment adherence [5] and are one of the main reasons for discontinuation [6]. Moreover, they significantly add to the costs of cancer treatment [2]. Therefore, factors that may contribute to the development and reduction of AEs warrant clinical attention.

Patients’ experience of AEs is susceptible to the nocebo effect [7]. Here, *nocebo effect* denotes any adverse response to a substance that cannot be attributed to its pharmacological effects. In its most tangible form, the nocebo effect occurs after exposure to an inert substance, such as a placebo pill [8]. Meta-analysis of clinical cancer trials showed that 10–60% of patients in the placebo-arms experienced AEs [9], which in fact mirrored those in the active drug arms. Arguably, these AEs are the result of patients’ negative expectations: for example, there is robust meta-analytic evidence that expectations are associated with the severity of post-chemotherapy nausea [10]. Such expectations may be evoked during informed consent [11]. The physiological effects of expectations have been underpinned by neurobiological correlates, primarily in nocebo pain modulation [12, 13].

Nocebo responses can also occur after intake of an active drug, which may be facilitated through misattribution of symptoms or patient expectations. Several studies have shown that patients report more AEs when they are informed about potential AEs [14–16]. However, many of the AEs from verum drugs are not attributable to pharmacological effects [7]. A potential mechanism of this is misattribution of pre-existing or unrelated symptoms [8]. In a general population study, the median number of symptoms (typically day-to-day ailments such as rash or bloating) reported in the past 7 days was five, with only 11% of participants reporting no symptoms [17–19]. These symptoms can be misattributed to new medications [18], especially when patients already experience many symptoms [20]. The nocebo effect can also lead to exacerbation of medication-specific AEs. A meta-analysis showed a medium-sized relationship between expecting and experiencing AEs of chemotherapy [21]. A further inducer of nocebo resembles the mechanism of classical conditioning: for example, prior exposure to chemotherapy increased the likelihood of pre-treatment nausea [21, 22].

An innovative means of reducing the nocebo effect suggested by Barsky and colleagues [8] is to inform patients about the nocebo effect. In theory, the awareness that not all symptoms are pharmacological effects of their therapy would allow patients to perceive symptoms as less threatening and therefore more tolerable [8]. Specifically, misattribution of non-specific symptoms may be reduced, and perceived control of symptoms as well as treatment expectations improved. Dysfunctional treatment expectations have been shown to influence adverse events of cancer treatment in breast cancer [23, 24]. In a first study [25], participants with self-reported chronic headache were recruited under the guise of participating in a clinical trial for a headache medication. All participants read a bogus medication leaflet before receiving a placebo pill, and half the patients’ leaflets included an explanation of the nocebo effect. Those who received the nocebo effect leaflet reported significantly less AEs than the control group (cf. [26]).

In summary, informing about the nocebo effect may be effective in reducing the experience of AEs. This type of intervention is fast, simple, cost-effective and ethically feasible; therefore, it could potentially serve as a component of adverse effect management. Moreover, it requires no alteration to clinical routine or informed consent. In this study, we therefore examine the efficacy of a nocebo education intervention in a clinical sample receiving verum medication. We hypothesized that patients undergoing chemotherapy for GI cancer would experience less AEs if they were informed about the nocebo effect. This patient population is exposed to considerable distress [27] and at risk for symptom misattribution: on top of possible symptoms from the underlying malignancy, two thirds of patients with GI cancer have comorbidities [28]. Suggesting that perceived AEs of chemotherapy are not solely caused by the drug itself may increase patients’ perceived self-efficacy in symptom management and reduce misattribution. To examine possible contributing factors to the effect of nocebo education, we assessed patients’ perceived control of AEs, their tendency to misattribute symptoms, compliance intention, attitude towards chemotherapy, clinician-rated toxicity and co-medication used to treat AEs. Moreover, we investigated whether optimized treatment expectations mediated hypothesized beneficial effects of nocebo education. As a monitoring information coping style is associated with a higher report of AEs [29], we also assessed the moderating effect of desire for information about AEs.

## Methods

### Procedures

The trial was approved by the University of Hamburg ethics committee (ID: 2015_03) and retrospectively registered on March 27th, 2018 in the German Clinical Trial Register (ID: DRKS00009501). The study was conducted in accordance with the declaration of Helsinki. Enrolment and follow-up assessments took place from 08/2015 to 05/2018 and 10/2015 to 09/2018, respectively.

Oncologists pre-screened patients for eligibility once they were indicated to receive chemotherapy. The study team informed patients verbally and in writing about the overarching goal of the study, namely, to gain insight into patients’ expectations about chemotherapy and QoL during treatment. All participants gave written informed consent prior to enrolment. They were then randomized 1:1 to the nocebo education session or the attention control session. Both sessions lasted 20–30 minutes and were conducted during or ≤ 24 hours before first chemotherapy. Both sessions were semi-manualized and conducted by a trained healthcare professional in an empathetic, patient-centred manner.

#### Nocebo education group

In this four-part session, patients learned about the concept of the nocebo effect. First, the healthcare professional asked patients about prior experiences with AEs of medications or other treatments. Second, the healthcare professional presented a case-example of a nocebo response [30]. Third, patients were handed a standardized information leaflet about the nocebo effect. Using the leaflet as a guide, they were encouraged to discuss about personal experiences with the nocebo effect. Fourth, patients reflected on how they might apply their knowledge of the nocebo effect to their chemotherapy AEs.

#### Attention control group

The objective of this group was to control for the non-specific effects of psychosocial interventions, i.e., patient-practitioner alliance and attention. Using an adapted version of the Functional Assessment of Cancer Therapy - General scale (FACT-G) [31], the healthcare professional interviewed the patient on physical, emotional and functional well-being beliefs as well as spirituality, and relationship to healthcare professionals. Session notes were later discarded.

Assessments were conducted immediately before (T1pre) and after (T1post) the intervention, and at 10 days (T2) and 12 weeks (T3) after onset of chemotherapy. Patients in the control group received the nocebo education leaflet by mail after their final assessment. Concomitant treatments, including psychosocial interventions, radiation therapy and medication, were permitted.

### Participants

Eligible patients were aged ≥18 years, fluent in German, chemotherapy-naïve and newly diagnosed with gastrointestinal cancer (i.e. oesophagus, stomach, pancreas, gallbladder, bile duct, small and large intestines, rectum, anus, and cancer of unknown primary with metastases in the gastrointestinal tract). Exclusion criteria were impaired capability of self-care (Eastern Co-Operative Oncology Group (ECOG) score ≥ 3), severe psychological disorder (schizophrenia, substance abuse, severe depression or severe anxiety disorder), acute medical condition, chronic skin or lung disease (or dyspnoea or rash before starting treatment) and treatment with epidermal growth factor receptor antibodies. As indicated in the study protocol [32], a sample of *n* = 90 was required to detect a between-group effect of medium size (Cohen’s *d* = 0.6), given 80% power and 5% alpha-error (two-tailed). Considering a potential drop-out rate of 10%, we aimed at including *N* = 100 patients.

### Randomization and blinding

We conducted a stratified randomization with block sizes of 2 and 4. Prior to first enrolment, a research assistant generated the allocation sequence using a computer program [33], and prepared sequentially numbered, opaque, sealed envelopes. The group allocation was stratified by distress, as it has been shown to impact the efficacy of psychosocial interventions for cancer patients [34]. Distress was assessed using a 10-point distress thermometer at T1pre (< 5 *low* vs. ≥ 5 *high*) [34]. Unaware of block-sizes, the trained healthcare professional performed the 1:1 group allocation after T1pre assessment by opening the envelope in front of the patient.

The trained healthcare professional performed both the randomization and intervention and was therefore not blinded. Patients were unaware of the specific research question and the content of the other intervention.

Except for manualized reminder calls for outstanding questionnaires in isolated cases, the study team did not interact with patients for the outcome assessments (data collection is further detailed in the study protocol [32]).

### Outcomes

Our primary outcome was group difference in AEs at 10 days (T2) and 12 weeks (T3) after onset of chemotherapy, assessed with the Generic Assessment of Side Effects (GASE), which demonstrated high internal consistency and validity [17]. Patients rated the severity of seven symptoms in the past 7 days from 0 *not present* to 10 *severe*. Four symptoms (nausea, vomiting, diarrhoea, and fatigue) were specific to the most common chemotherapeutics used to treat gastrointestinal tumours (mainly fluoropyrimidines and/or platinum agents) [35–39]. Three symptoms (headache, shortness-of-breath and rash) were non-specific to chemotherapy [35–39]. The item range was increased (original GASE: 0–3) in the interest of higher outcome sensitivity. A further item assessed global rating of adverse events (“Overall, how strongly did you experience adverse effects in the past 7 days?”) from 0 *not present* to 10 *severe*.

Secondary outcomes included perceived control of AEs [32], misattribution tendency [32], use of co-medication to treat AEs (yes/no), and clinician-rated toxicity (Version 4.03 [40]) at T2 and T3. Compliance intention and attitude towards chemotherapy were assessed at T2. Patients’ ability to control each symptom was assessed on a scale from 0 *not at all* to 10 *completely* using the adapted GASE-Coping, which has been previously used in a study with breast cancer patients [41]. We calculated a mean control score for patients who experienced at least one AE. Misattribution items were adapted from the GASE [41]. For each symptom, patients indicated to which degree they attribute it to the chemotherapy (from 0 *not at all* to 10 *completely*). To obtain misattribution tendency, we computed a mean across attribution tendencies of the non-specific AEs headache, shortness-of-breath, and rash*.* Compliance intention was assessed with two items: ﻿“How certain are you about completing the chemotherapy?” rated from 0 *not at all* to 10 *very* and “How high is the probability that you might terminate the chemotherapy prematurely on your own account?” from 0 to 100%. We re-scaled the latter item and calculated a mean score. Patients’ attitude towards chemotherapy in general was evaluated with the item “How would you describe your attitude towards chemotherapy?” from 0 *very negative* to 10 *very positive.* Clinician-rated toxicity (i.e., AEs of chemotherapy) of the seven AEs assessed in self-rating were retrieved from patients’ medical records, as routinely assessed by the attending physician before every cycle using the Common Terminology Criteria for Adverse Events (CTCAE) Version 4.03 [40], a standardized system which grades adverse events according to organ specific parameters. For comparability with patients’ self-rating of AEs, we used the gradings recorded at the consultations closest to T2 and T3.

### Further assessments

Patients’ expectations of the severity (AE expectations; “How much to you expect to experience [symptom]?”) and expected control of each AE (control expectations; “How much to you expect to be able to influence [symptom]?”) were assessed at T1pre and T1post. Items were rated on a scale of 0 *not at all* to 10 *completely*. Cronbach’s alpha indicated good internal consistency (AE expectations: α = .89–.91; control expectations: α = .88–.91).

Sociodemographic data, distress level, tumour site, chronic somatic diseases, and desire for information were self-reported at T1pre. Tumour stage (UICC [42]), treatment aim and chemotherapy regimens at T1pre and tumour progression (yes/no) at T2 and T3 were retrieved from medical records. At T1post, patients evaluated the relevance of the respective intervention and whether they would recommend it to other patients (0 *not at all* to 10 *completely*). As a manipulation check, the nocebo education group was asked to give free-text descriptions of the nocebo effect at T1post and T2.

### Statistical analysis

All analyses were conducted with the intention-to-treat sample. Among completed questionnaires, missing values ranged between 1 and 6.3% per item. Data were imputed using multiple imputation and the fully conditional specification method. Both death and discontinuation of chemotherapy were included as indicators [43]. We generated 15 imputed datasets and pooled parameters according to Rubin’s rule [44]. Adjusted degrees of freedom were computed by hand and in alignment with the R package mice’s procedures [45]. Co-medication to treat AEs (yes/no) was imputed from medical records. CTCAE data were missing for 27 patients at T2 and 40 patients at T3, and were not imputed. SPSS version 25.0 [46] was used for data analyses and imputation.

We computed linear mixed models for repeated measures using restricted maximum-likelihood estimation and a variance component matrix type for our primary outcome AEs and our secondary outcomes control of AEs and misattribution tendency. The assessment timepoints T2 and T3 (level-1) were nested within patients (level-2). Fixed effects included Group, Time, Group x Time, Distress, and Cancer Staging. The intercept was included as a random effect. Group differences were examined via pairwise comparisons. Assumptions of linear models were checked prior to analyses [47]. The outcomes specific AEs, non-specific AEs, control of AEs, and misattribution tendency were right-skewed and therefore transformed to meet assumptions of residual normality and homoscedasticity. A square root transformation (formula: √[X + 1]) was chosen based on visual examination and after Kirk’s [48] systematic approach.

We conducted regression analyses to examine the group difference in attitude towards chemotherapy (linear regression), compliance intention (Poisson regression), and co-medication to treat AEs (yes/no; logistic regression). In all multivariate analyses, the stratum distress, cancer staging, and (if existent) the baseline of the respective outcome variable, were included as covariates [32].

We calculated risk ratios for experiencing at least one AE based on the CTCAE.

We hypothesized that the effect of Group on AEs is mediated through changes in expectations. We calculated two models: group as predictor (X), AEs at T2 and T3 as outcomes (Y), and change in expected AEs (model 1; M_1_) or change in expected control of AEs (model 2; M_2_) as the mediator. We obtained path a via regression analyses, and paths b and c’ via linear mixed models [49]. All effects were standardized. Mediation effects were examined using Monte Carlo simulations with 20,000 repetitions [50, 51]; mediation was established if the confidence interval around the indirect effect did not contain zero.

Lastly, desire for information about AEs was examined as a moderator of the primary outcome by including the desire for information and desire for information x Group as additional fixed effects in the linear mixed models.

Sensitivity analyses of the primary outcome can be found in Supplementary Material A.

## Results

### Patient flow

**Fig. 1:**
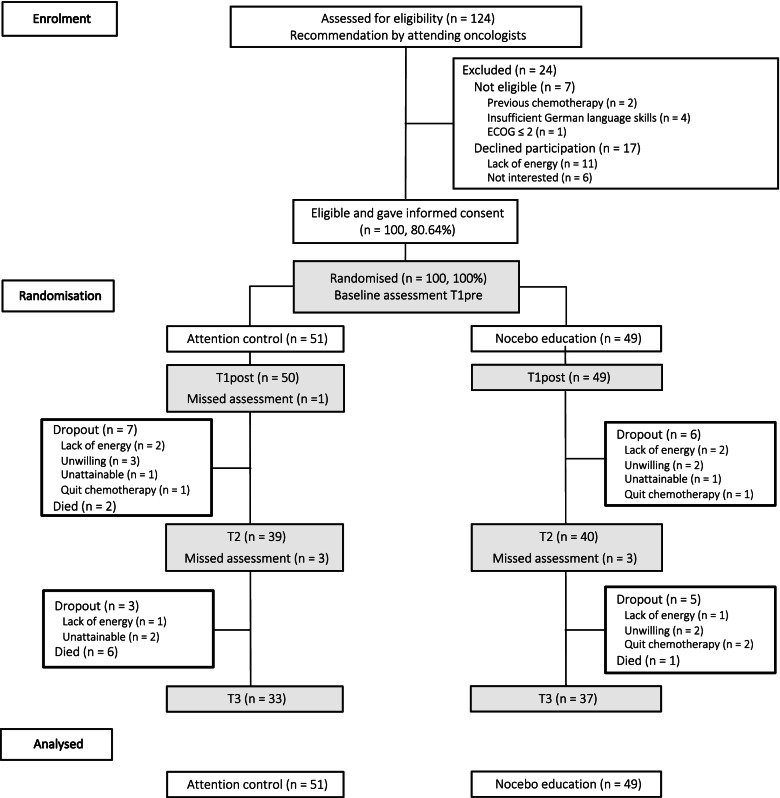
CONSORT diagram. ECOG = Eastern Co-operative Oncology Group; values ≤ 2 indicate limited capability of self-care. Assessment points are shaded. T1pre and T1post = immediately pre- and post-intervention, T2 = 10 days after onset of chemotherapy and T3 = 12 weeks after onset of chemotherapy. Missed assessment = patients who missed the respective assessment but remained enrolled. One patient in the attention control group discontinued chemotherapy but completed T3

Of all patients pre-screened by their oncologist, *n* = 124 were referred for eligibility assessment (Fig. 1). Thereof, *N* = 100 participants were randomized into the nocebo education (*n* = 49) and attention control (*n* = 51) groups. Participants received the respective intervention during the first course of chemotherapy, except for three who received it prior. The dropout rate was 30%; by T3, *n* = 12 (24.4%) and *n* = 18 (35.3%) patients in the nocebo education and control group were lost. The range of completion was 4–72 days after onset of chemotherapy (*M* = 19.5, *SD* = 13.22) for T2 (scheduled: 10 days) and 73–225 days (*M* = 110.1, *SD* = 31.54) for T3 (scheduled: 12 weeks i.e., 84 days). Exploratory analysis revealed no correlation between completion time and the main outcome (sum score of AEs at T2 and T3; *p*s > .11).

Patient characteristics are detailed in Table 1. To estimate comparability of chemotherapy regimens and expected adverse events between groups, we calculated and found that the number of patients who received platin-based chemotherapy did not differ between groups (*X*^*2*^ (1, *N* = 100) = 0.433, *p* = 0.51).

**Table 1:** Sample characteristics at baseline

	Total Sample (*N* = 100)	EG (*n* = 49)		CG (*n* = 51)	
	*N*^*a*^	*N*	%	*N*	%
Age, years (*M*, *SD*)	60.22 (11.45)	58.53	12.39	61.84	10.20
Gender (female)	35	15	30.6	20	39.2
Education					
≤ 10 year of school	56	25	51	31	60.8
13 years of school	23	12	24.5	11	21.6
University degree	21	12	24.5	9	17.6
Employment status					
Employed	51	27	55.1	24	47.1
Freelancer	20	8	16.3	12	23.5
Homemaker	5	2	4.1	3	5.9
Unemployed	2	2	4.1	0	0
Pensioner	22	10	20.4	12	23.5
Location					
University Clinic Hamburg-Eppendorf	91	45	91.8	46	90.2
Cooperating practice	9	4	8.2	5	9.8
Healthcare professional^b^					
BSc Psychologist (female)	35	19	37.3	16	45.7
Medical doctoral candidate (male)	65	32	62.7	33	32.7
Cancer Staging (UICC)					
l	4	2	4.1	2	3.9
ll	5	3	6.1	2	3.9
lll	37	14	28.6	23	45.1
IV	54	30	61.2	24	47.1
Type of cancer					
Upper gastrointestinal tract	31	10	20.4	21	41.2
Lower gastrointestinal tract	35	22	44.9	13	25.5
Gallbladder & biliary tract	8	4	8.2	4	7.8
Cancer of unknown primary	3	2	4.1	1	2.0
Liver	1	1	2.0	1	2.0
Pancreas	10	10	20.4	11	21.6
Type of chemotherapy					
Adjuvant	25	14	28.6	11	21.6
Neoadjuvant	24	9	18.4	15	29.4
Palliative	49	24	49.0	25	49.0
Additive	2	2	4.1	0	0
Additional radiation therapy	12	5	10.2	7	13.7
First-line chemotherapy regimen					
Fluoropyrimidine/ platin doublet^c^	37	18	36.7	19	37.2
Fluoropyrimidine/ platin triplet^d^	14	5	10.2	9	17.7
Platin-based doublet^e^	25	16	32.7	9	17.7
Other doublets^f^	7	2	4.1	5	9.8
Monotherapy^g^	11	7	14.3	4	7.8
Missing information	6	1	2.0	5	9.8
Physical comorbidity present	40	17	34.7	23	45.1
High distress (≥ 5)^h^	71	35	71.4	36	70.6
Distress (*M, SD*)^i^	5.74 (2.89)	5.75	2.83	5.73	2.98
Compliance intention	9.09 (1.41)	9.01	1.54	9.17	1.29
Attitude towards chemotherapy (*M, SD*)^j^	7.85 (2.19)	7.78	2.32	7.92	2.07
Perceived efficacy of the chemotherapy (*M, SD*)^k^	8.72 (1.52)	8.67	1.62	8.78	1.44
Desire for information about AEs (*M*, *SD*)^l^	6.92 (3.01)	6.98	2.93	6.86	3.11
Expected AEs (*M, SD*)^l^	3.85 (1.86)	3.94	1.73	3.75	2.00
Expected control of AEs (*M, SD*)^l^	4.72 (1.95)	4.86	1.94	4.59	1.97

*AEs* adverse events, *CG* attention control group, *EG* nocebo education group, *UICC* Union for International Cancer Control

^a^As overall sample size is *N* = 100, percentages equal numbers

^b^The healthcare professional delivered the nocebo education or conducted the quality of life interview in the attention control group

^c^FOLFOX (leucovorin, 5-fluorouracil & oxaliplatin), FUFOX (high dosage 5-fluorouracil, folic acid & oxaliplatin), CAPOX (capecitabine + oxaliplatin), FLO (5-fluorouracil, leucovorin & oxaliplatin)

^d^FOLFIRINOX (﻿5-fluorouracil, irinotecan & oxaliplatin), FLOT (5-fluorouracil, leucovorin, oxaliplatin & docetaxel)

^e^carboplatin + etoposide, carboplatin + taxane, GEM (gemcitabine) + cisplatin, cisplatin + CAP (capecitabine), 5-FU (fluorouracil) + cisplatin

^f^FOLFIRI (5-FU, folic acid & irinotecan), CAP / 5-FU + mitomycin, GEM + taxane

^g^5-FU, GEM, CAP

^h^Groups were stratified for distress

^i^Scale ranges from 1 to 10

^j^Scale ranges from 0 to 10, higher values indicate a more positive attitude

^k^Scale ranges from 0 to 10, higher values indicate believing in the efficacy

^l^Indicated for *n* = 99 patients at T1post

Most patients received either postoperative chemotherapy (≥ 3 months, ≤ 6 months) or palliative chemotherapy (treatment applied until progression or intolerability). Only 24 patients received neoadjuvant chemotherapy, mostly for either pancreatic or locally advanced colorectal cancer (≥ 3 months). Neoadjuvant treatment for less than 3 months was applied in 8 patients with esophagogastric cancer, however due to delays, the interval between last chemotherapy and T3 was less than 3 weeks in all these patients.

### Primary outcome: adverse events

**Fig. 2:**
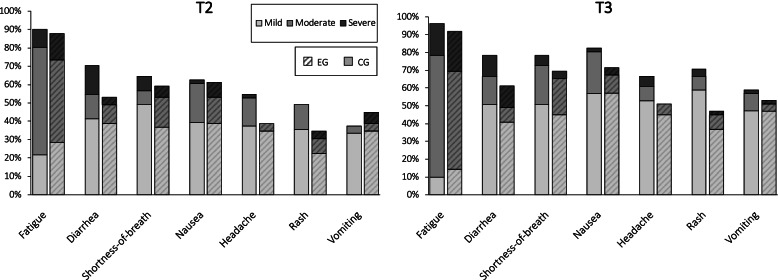
Severity and frequency of AEs at ten days and 12 weeks after first dose of chemotherapy (imputed data). Symptom severity was graded into 1–3 *mild*, 4–7 *moderate* and 8–10 *severe*. Specific adverse events: fatigue, diarrhoea, nausea and vomiting; non-specific adverse events: shortness-of-breath, headache and rash. CG = attention control group (*n* = 51); EG = nocebo education group (*n* = 49)

At T2, four patients and at T3, one patient indicated no AEs. Among patients who did, the mean sum score of all seven adverse events was 14.83 (range: 1–36) at T2 and 17.61 (range: 3–44) at T3. At T2, 22 and at T3, 12 patients reported no non-specific AEs. The global rating of adverse events had a moderately positive correlation with total AEs (specific + non-specific) at T2 (*r* = 0.62, *p* < .001) and T3 (*r* = 0.56, *p* < .001). Type and severity of self-reported AEs are detailed in Fig. 2.

At T3, AEs in the nocebo education group were significantly lower, by 4.04 points (*SE* = 1.69), than in the control group (Table 2). Similarly, square root transformed non-specific AEs were significantly lower in the nocebo education group at T3 by 0.39 points (*SE* = 0.18). Group differences in trend were found for both square-root transformed specific AEs and the global AE scale at T3. At T2, AEs did not differ between groups.

**Table 2:** Group differences in adverse events, control of adverse events, and misattribution tendency

	T2							T3						
	CG			EG			Group Comparison	CG			EG			Group Comparison
	*M*	*SE*	*N*	*M*	*SE*	*N*		*M*	*SE*	N	*M*	*SE*	N	
Total AEs	14.87	1.19	51	13.58	1.20	49	Mean difference: 1.30, 95% CI [−2.00, 4.59], *Wald* = 0.77, *df* = 87, *p* = .44, *d* = 0.15	19.41	1.19	51	15.37	1.21	49	**Mean difference: 4.04, 95% CI [0.72, 7.36],** ***Wald*** **= 2.39,** ***df*** **= 86,** ***p*** **= .02,** ***d*** **= 0.48**
Specific AEs	3.14	0.14		3.10	0.14		Mean difference: 0.04, 95% CI [−0.34, 0.41], *Wald* = 0.19, *df* = 88, *p* = .85, *d* = 0.04	3.62	0.14		3.26	0.14		Mean difference: 0.36, 95% CI [−0.02, 0.74], *Wald* = 1.84, *df* = 85, *p* = .07, *d* = 0.37
*Original scale*	8.85			8.62				12.12			9.65			
Non-Specific AEs	2.29	0.13		2.05	0.13		Mean difference: 0.24, 95% CI [−0.11, 0.59], *Wald* = 1.35, *df* = 82, *p* = .18, *d* = 0.27	2.61	0.12		2.22	0.13		**Mean difference: 0.39, 95% CI [0.04, 0.73],** ***Wald*** **= 2.19,** ***df*** **= 83,** ***p*** **= .03,** ***d*** **= 0.44**
*Original scale*	4.26			3.22				5.81			3.94			
Global AE scale	4.43	0.37	51	3.90	0.37	49	Mean difference: 0.53, 95% CI [−0.49, 1.56], *Wald* = 1.02, *df* = 86, *p* = 0.31, *d* = 0.20	5.45	0.37	51	4.32	0.39	49	Mean difference: 1.01, 95% CI [−0.02, 2.05], *Wald* = 1.92, *df* = 84, *p* = .06, *d* = 0.38
Control of AEs	1.88	0.07	48	1.93	0.07	48	Mean difference: −0.05, 95% CI [−0.23, 0.14], *Wald* = 0.51, *df* = 83, *p* = .61, *d* = 0.10	1.76	0.06	51	1.77	0.07	48	Mean difference: − 0.01, 95% CI [−0.19, 0.17], *Wald* = 0.07, *df* = 87, *p* = .95, *d* = 0.01
*Original scale*	2.53			2.71				2.11			2.13			
Misattribution tendency	2.08	0.08	42	1.89	0.08	36	Mean difference: 0.19, 95% CI [−0.04, 0.42], *Wald* = 1.65, *df* = 58, *p* = .10, *d* = 0.38	2.21	0.07	47	2.08	0.08	41	Mean difference: 0.13, 95% CI [−0.07, 0.34], *Wald* = 1.27, *df* = 72, *p* = .21, *d* = 0.27
*Original scale*	3.31			2.55				3.89			3.33			

Pooled means and standard errors of linear mixed models after adjusting for distress and cancer staging. The primary outcome, total adverse events (AEs), is a sum-score of 7 symptoms (4 specific, 3 non-specific) each rated on a 10-point severity-scale (range: 0 to 70). Specific AEs (range: 0–40) and non-specific AEs (range: 0–30) are its subscales. The global AE scale ranged from 0 to 10. Control of adverse events (range: 0 to 10) was computed for patients who reported at least one AE. Misattribution tendency (range: 0 to 10) was computed for patients who reported non-specific AEs. Means on the original scales were obtained by back transforming the estimates. Significant differences were indicated in bold. Primary outcomes are shaded

*AE* adverse event, *T2* 10 days after onset of chemotherapy, *T3* 12 weeks after onset of chemotherapy, *CG* attention control group, *EG* nocebo education group, *CI* Confidence interval, *d* Cohen’s d

### Secondary outcomes

On average, patients rated their ability to control AEs and their misattribution tendency as low (Table 2). Linear mixed models indicated no significant group differences in perceived control of AEs and misattribution tendency (Table 2).

Patients’ overall attitude towards their chemotherapy was positive (*M* = 7.45, *SD* = 2.03) and compliance intention was very high (*M* = 8.76, *SD* = 1.41), with 45% of patients indicating a maximum score of 10. Both variables did not differ by group (attitude towards chemotherapy: β = 0.06, 95% CI [−0.34; 0.46], *p* = .79; compliance intention: *OR* = 0.76, 95% CI [0.51; 1.19], *p* = .24).

At T2 *n* = 52 (of *n* = 82; 63.4%) and at and T3, *n* = 54 (of *n* = 83, 65.0%) patients reported using co-medication to treat AEs, with no significant group differences (T2: *OR* = 1.98, 95% CI [0.77, 5.12], *p* = .16, *Nagelkerke’s R*^*2*^ = .09; percentage of correctly predicted cases: 68.3%; T3: *OR* = 0.73, 95% CI [0.29, 1.85], *p* = .51, *Nagelkerke’s R*^*2*^ = .06; percentage of correctly predicted cases: 66.3%).

Descriptive statistics of clinician-rated AEs according to CTCAE [40] are given in Table 3. The risk ratio for developing at least one AE when allocated to nocebo education vs. control group was 1.14 at T2 (95% CI [0.73, 1.78], *p* = .57), and 1.25 at T3 (95% CI [0.72; 2.16], *p* = .43).

**Table 3:** Clinician-rated adverse events of chemotherapy at T2 and T3 according to Common Terminology Criteria for Adverse Events

	T2							T3						
	EG (*n* = 38)			CG (*n* = 35)			Sum	EG (*n* = 31)			CG (*n* = 29)			Sum
	G1	G2	G3	G1	G2	G3		G1	G2	G3	G1	G2	G3	
Nausea	6	6	0	4	1	0	17	4	2	0	3	0	1	10
Vomiting	0	2	0	3	0	0	19	0	0	0	1	0	1	2
Diarrhoea	4	2	0	7	2	0	15	5	1	0	2	1	0	9
Fatigue	4	6	0	1	1	0	12	4	5	0	3	1	1	14
Headache	0	0	0	0	1	0	1	0	0	0	1	0	0	1
Shortness-of-breath	0	1	0	0	0	0	1	2	0	0	1	0	0	3
Rash	2	0	0	0	0	0	2	1	0	0	0	1	0	2
	≥1 AE: *n* = 21			≥1 AE: *n* = 17				≥1 AE: *n* = 16			≥1 AE: *n* = 12			

*AE *adverse events, *CG *attention control group, *EG *nocebo education group, *T2 *10 days after onset of chemotherapy; *T3 *12 weeks after onset of chemotherapy. *G1 *Grade 1 “Mild; asymptomatic or mild symptoms; clinical or diagnostic observations only; intervention not indicated”, *G2 *Grade 2 “Moderate; minimal, local or non-invasive intervention indicated; limiting age-appropriate instrumental activities of daily living, *G3 *Grade 3 “Severe or medically significant but not immediately life-threatening; hospitalization or prolongation of hospitalization indicated; disabling; limiting self-care activities of daily living (defined by the National Institutes of Health, National Cancer Institute, 2009)

### Mechanisms of change

**Fig. 3:**
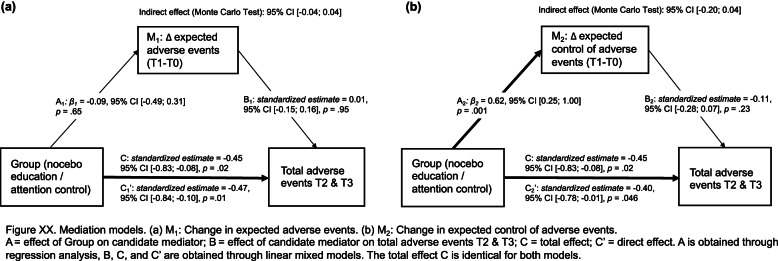
Mediation models. **a** M1: Change in expected adverse events. **b** M2: Change in expected control of adverse events. A = effect of Group on candidate mediator; B = effect of candidate mediator on total adverse events T2 & T3; C = total effect; C′ = direct effect. A is obtained through regression analysis, B, C, and C′ are obtained through linear mixed models. The total effect C is identical for both models

Figure 3 shows the results of the two mediation models. Neither of our two hypothesized mediators were found to explain the effect of the group on our primary outcome total AEs at T2 and T3. The Monte Carlo test of mediation indicated no indirect effect of change in expected AEs (M_1_; 95% CI [− 0.04; 0.04]), nor of change in expected control of AEs (M_2_: 95% CI [− 0.20; 0.04]).

### Moderator of the intervention

The interaction effect of Group x Desire for information about AEs was not significant in the linear mixed model (Y = total AEs), indicating no moderating effect of desire for information about AEs on total AEs (estimate: 0.06, 95% CI [− 0.43; 0.54], *p* = .82).

### Evaluation of the intervention

Patients in both groups rated the conversation as highly relevant (nocebo education group: *M* = 8.08, *SD* = 1.90; control group: *M* = 7.30, *SD* = 2.19; range: 0–10) and indicated highly recommending it to other patients (nocebo education group: *M* = 8.94, *SD* = 1.44; control group: *M* = 8.48, *SD* = 1.87; range: 0–10).

## Discussion

AEs of chemotherapy are susceptible to the nocebo effect [21]. In the present study, we tested whether a nocebo education intervention could reduce AEs of chemotherapy in patients with GI cancer.

For our primary outcome, we found that 12 weeks (T3) after onset of chemotherapy, patients in the nocebo education group experienced significantly less AEs than the control group; specifically, they had less non-specific AEs and a trend towards less specific AEs. At 10-days (T2), there was no group difference. We found no group differences in control of AEs, misattribution of non-specific AEs to chemotherapy, attitude towards chemotherapy, compliance intention, use of co-medication to treat AEs, or risk of developing at least one AE. Further, information coping style did not influence the group difference in AEs.

### Adverse events

This is the first study to show that the AEs of a medication can be reduced by educating patients about the nocebo effect, at a moderate effect size. Thereby, it confirms the clinical transferability of this concept from findings of two prior studies which experimentally induced symptoms that were subsequently reduced by nocebo education [25, 26].

The group difference in AEs was mainly attributable to a reduction in non-specific AEs (headache, shortness-of-breath, and rash) in the nocebo education group. As we expected, the prevalence of non-specific AEs surpassed rates defined in chemotherapy drug trials [35–39]. In such trials, AEs are rated by blinded physicians or nurse practitioners and rated as such if they significantly surpass the AEs observed in the placebo arm of the trial. In contrast, patients who are reporting on their self-perceived symptoms will commonly indicate common ailments known to be highly prevalent in the general population [18]. Therefore, the discrepancy between self-reported and observed AEs is often considerable [52, 53].

A plausible explanation for the reduction of non-specific AEs is a learning effect [54]: with each dose of chemotherapy, the causal relationship between chemotherapy and specific AEs is reinforced. This may have helped patients to differentiate them from non-specific AEs. The intervention aimed at teaching patients to identify non-specific AEs as potentially nocebogenic, and therefore less threatening, which may have decreased patients’ perceived severity. This learning process may have taken some time, potentially explaining why the group difference was detected after 12-weeks (T3) but not after 10-days (T2). There was a trend towards reduction of specific AEs at T3 as well, yet the simultaneous, powerful conditioning effect from preceding chemotherapy doses [8, 21, 55] may have amplified specific AEs to the extent that they were less cognitively mutable than non-specific AEs [56]. Conversely, in patients whose preemptive co-medication – as is standard protocol for the specific AE nausea [57] – was effective, the nocebo education session was perhaps not capable of further AE reduction. This is not however plausible for fatigue, which has no gold-standard pharmacological treatment [58]. In summary, specific AEs may be less modifiable than non-specific AEs because pronounced specific AEs are subject to conditioning effects, whereas medication-controlled AEs may already be sufficiently controlled.

Other trials aimed at reducing nocebo effects of verum medications have used positive framing methods. A recent review [59] shows that only one out of three studies which applied framing to AEs of a medication achieved a significant reduction of 2 out of 12 listed AEs [60]. This underlines that medication AEs are difficult to modify, emphasizing the relative impact of our findings. Since our research is novel in its method of AE modification as applied to verum medication, our results require replication.

In the context of psychosocial interventions for GI cancer patients, our intervention demonstrates favourable results. Mosher et al. [61] conducted a systematic review of 14 studies using interventions such as education, supportive care and relaxation in patients with colorectal cancer. Six of the eight interventions with QoL outcomes that included disease or treatment specific symptoms produced no effect [61]. One study in which patients were provided with regular home visits for informational and emotional support compared to treatment as usual showed a reduction in fatigue (*p* = .048), but not other symptoms such as diarrhoea or shortness-of-breath [62]. Likewise, training in progressive muscle relaxation improved overall colorectal cancer-related QoL (*p* < .001) and physical health specifically (*p* < .01) [63]. Notably, both these interventions were considerably longer than ours yet their effects, unlike ours, were not controlled for attentiveness from the delivering healthcare professional. Findings from two further studies including a sample of primarily GI cancer [64] and hepatocellular-, gallbladder or cholangiocellular carcinoma [65] patients have the same trajectory: individually tailored psychotherapy sessions caused clinically relevant reductions in AEs [65] and a web-based collaborative care intervention with fortnightly follow-ups likewise produced small to medium effect sized improvements on pain and QoL [64]. Therefore, we conclude that our intervention was effective and efficient compared to other psychosocial interventions.

The prevalence of self-reported AEs resembles self-report from *n* = 142 colorectal cancer patients receiving chemotherapy; the rank order of fatigue, diarrhoea, dyspnoea, rash and vomiting also mirror [1]. In a further study of self-report symptom prevalence in colorectal cancer patients, fatigue was also the most commonly, and rash and vomiting the most seldomly reported of the symptoms that we analysed [66]. The high prevalence of fatigue reflects the lack of effective pharmacological treatment, whereas vomiting appears effectively controlled with pre-treatment antiemetics. The overall higher prevalence of symptoms at T3 than at T2 also corresponds to self-report findings by which chemotherapy AEs accumulate [1].

Our results demonstrate the clinical feasibility and the efficacy of the nocebo intervention in reducing AEs with clinically relevant, moderate to large effects sizes in comparison to a psychological control intervention. Further studies are needed to analyse potential action mechanisms of this treatment option.

### Secondary outcomes

Across groups, patients rated their ability to control AEs at 10-days and 12-weeks follow-up as low. Good strategies for symptom control lead to less AEs and better health-related QoL [67, 68], hence the large amount of cancer care interventions with this target ([69–71]). Improvement in perceived control has likewise been proposed as a pathway of AE reduction through nocebo education [72]. Perhaps no group difference in control of AEs emerged in our study because the intervention did not target symptom control strategies.

Misattribution of non-specific AEs to chemotherapy was also low in both groups. One explanation would be to posit that all patients were all patients were well informed by their attending physicians about which AEs to expect, and therefore were not prone to misattribution. There are, however, several other aspects to consider. Prior findings on the impact of symptom misattribution focus on the immediate AEs after one-time, inert substance intake [20, 25], the psychological mechanism of which might be inapplicable to repeated chemotherapy. A positive attitude towards medicines, as displayed by our sample towards chemotherapy, has been shown to reduce misattribution; likewise, prior knowledge about specific chemotherapy AEs may have helped patients to identify non-specific AEs as such. To summarize, there are several potential explanations for the absence of symptom misattribution, preventing conclusions about the interplay of nocebo education and symptom misattribution in the current study.

We also analysed several clinically relevant outcomes. Compliance intention at 10-days was high across groups and, predictably, dependent upon its baseline level. We regarded compliance intention as a proxy for medication adherence. Our findings align with clinical adherence rates, with 78% of 3193 above 66-year-olds completing their chemotherapies for stage 3 colon carcinoma [73].

Group allocation was not predictive of whether or not patients reported using co-medication to treat AEs. The interpretability of this finding is limited by the large number of missing values on self-reports assessing co-medication use, which in turn were imputed from medical records. As these document the clinical oncologists’ prescriptions, but not medications prescribed by general practitioners or obtained over-the-counter (e.g., loperamide for diarrhoea or dimenhydrinate for nausea), they only indirectly reflect the actual co-medication taken. Further, we did not assess dosage information so as to minimize the burden on patients, therefore possible dose reductions in response to lessened AEs could not be detected.

Unlike the self-reported total AEs, clinician-rated toxicity of AEs did not differ between groups. Disparity between self-and clinician-rated AEs of chemotherapy is well established [52, 53], and researchers argue that self-rated toxicity deserves clinical attention as it more closely reflects patients’ QoL [2, 52].

### Further assessments

Desire for information about AEs did not moderate the intervention effect. Since a monitoring coping style (i.e., attending to and seeking information on symptoms) is associated with more AEs after admission of verum medications [29] as well as placebos [25], we assumed the intervention would buffer the influence of desire for information about AEs. Yet in line with recent findings [25], this relationship was not confirmed.

Patients rated both the intervention and attention control interview as highly relevant and indicated they would recommend it to other patients. Patients’ free-text descriptions of the nocebo effect at 10-days, such as: “a self-fulfilling prophecy”, verified that the majority gained a solid understanding. Given that the nocebo education information was only provided once and patients were under strain of their cancer symptoms, we consider this feedback positive.

### Strengths

In designing this study, our decisions were led by pragmatism in order to maximize the clinical validity of our results [74]. This strategy included recruitment through usual care, clinically determined in- and exclusion criteria, the liberality of which resulted in a heterogeneous and highly burdened sample with almost half of patients receiving palliative care, as well as an intervention that fit almost seamlessly into clinical routine. In effect, our findings have high external validity.

### Limitations

We did not assess baseline levels of the seven analysed AEs, so it is unclear to which extent pre-existing symptoms influenced our results. For example, higher non-specific symptom burden at baseline can exacerbate nocebogenic AEs after starting a medication [20, 25], and symptoms of the underlying malignancy can mirror those of chemotherapy [75, 76], therefore the former could have been misattributed to the latter [77], reducing the observed intervention effect. The same effect may have occurred within the study period in patients who received concurrent radiation therapy, in that radiation sunburn may have been misinterpreted as rash from chemotherapy.

There was a high variance in the actual completion times of T2 and T3, limiting the interpretation of the intervention effect over time. We attribute this to the high burden of disease in our sample as well as delays due to lost questionnaires.

We did not control for the AE information patients received from their attending physician during informed consent, which can vary considerably [78] and have a substantial effect on patients’ experience of AEs [72]. Finally, several items used were self-developed and not subjected to prior psychometric evaluation, limiting their comparability.

## Conclusions

In severely ill, burdened patients, we showed that a single education session about the nocebo effect reduced the AEs of chemotherapy. In addition to specific AEs, many patients in our sample suffered from pharmacologically unlikely non-specific AEs, emphasizing that the latter must not be underestimated in their potential negative impact to patients’ health. While our results are promising, they require replication to expand our current knowledge of modifying nocebo responses to medications by means of psychoeducation and cognitive reappraisal. Our intervention was integrated seamlessly into clinical routine and has the conceptual flexibility to be applied in various clinical settings. Nocebo education by no means replaces treatment such as co-medication for severe AEs; it rather serves as a low-level, patient empowering, supplementary measure. The potential of this line of research is that knowledge of the nocebo effect and coping with negative expectations becomes inherent to what we consider an informed patient.

## Supplementary Information

**Additional file 1: Supplementary Material A.** Sensitivity analysis. Sensitivity analysis of primary outcomes.

## Data Availability

The datasets used and/or analysed during the current study are available from the corresponding author on reasonable request.

## Abbreviations

AEs: adverse events
CG: attention control group
CI: confidence interval
CTCAE: Common Terminology Criteria for Adverse Events
ECOG: Eastern Co-Operative Oncology Group
EG: nocebo education group
FACT-G: Functional Assessment of Cancer Therapy - General
GASE: Generic Assessment of Side Effects
GI: gastrointestinal
PP: per-protocol
QoL: quality of life
UICC: Union for International Cancer Control
