# Supplementary material for: Preventing adverse events of chemotherapy for gastrointestinal cancer by educating patients about the nocebo effect: a randomized-controlled trial
Source: BMC Cancer. 2022 Sep 23;22:1008. doi: 10.1186/s12885-022-10089-2 (PMC9502603; doi:10.1186/s12885-022-10089-2)
Supplement: Supplementary file 1 — Additional file 1: Supplementary Material A. Sensitivity analysis. Sensitivity analysis of primary outcomes. [file 12885_2022_10089_MOESM1_ESM.docx]

**Supplementary Material A**

**Sensitivity analysis**

Sensitivity analyses were conducted with (1) the per-protocol (PP) sample, (2) after exclusion of patients who described the nocebo effect incorrectly, and (3) after exclusion of patients with cancer progression during the study. As all patients underwent the intervention session, protocol compliance was defined as having completed at least one follow-up assessment, non-discontinuation of chemotherapy, and being alive at assessment time.

To obtain the PP sample, we excluded nine patients who died, five patients who discontinued their chemotherapy (EG: n = 3, CG: n = 2) and another 11 patients who did not complete any follow-up questionnaires, resulting in *n* = 35 and *n* = 40 patients in the CG and EG. There were no differences between the results using the ITT or the PP sample (Table S1). After exclusion of patients who did not grasp the concept of the nocebo effect (n = 3), the intervention effect appeared more pronounced than in the ITT sample: at T3, there were less specific AEs (square root transformed) in the EG than in the CG (mean difference = 0.40, *SE* = 0.04, *p* = .048, Cohen’s *d* = 0.41). The same trend was observed when patients with tumour progression within the study period (CG: *n* = 4, EG: *n* = 1) were excluded, in that all four measures of AEs were lower in the EG. That is, in contrast to ITT analyses, specific AEs (square root transformed) differed at T3 by 0.43 points (*SE* = 0.36, *p* = .03, *Cohen’s d* = 0.45), and overall AEs differed by 1.26 points (*SE* = 0.27, *p* = .02, *Cohen’s d* = 0.46).

Table S1. Sensitivity analyses: Group differences by adverse effects

|  | **T2** | | | | | | | **T3** | | | | | | | | | | | | | |  |
| --- | --- | --- | --- | --- | --- | --- | --- | --- | --- | --- | --- | --- | --- | --- | --- | --- | --- | --- | --- | --- | --- | --- |
|  | CG | | | EG | | | Group Comparison | | CG | | | | | EG | | | | | | Group Comparison | | |
|  | *M* | *SE* | *N* | *M* | *SE* | *N* |  |  | *M* | *SE* | | | N | *M* | | *SE* | | N | |  | | |
| Per-Protocol sample |  |  |  |  |  |  |  | |  | |  |  | | |  | |  | |  | |  |  |
| AEs | 15.36 | 1.50 | 35 | 13.76 | 1.40 | 40 | Mean difference: 1.60, 95% CI [-2.43, 5.62], *Wald* = 0.77, *df* = 66, *p* = .44, *d* = 0.18 | | 19.89 | | 1.50 | 35 | | | 15.07 | | 1.42 | | 40 | | **Mean difference: 4.82, 95% CI [0.78, 8.87], *Wald* = 2.34, *df* = 65, *p* = .02, *d* = 0.54** |  |
| Specific AEs | 3.14 | 0.17 |  | 3.12 | 0.16 |  | Mean difference: 0.02, 95% CI [-0.44, 0.49], *Wald* = 0.10, *df* = 66, *p* = .92, *d* = 0.02 | | 3.61 | | 0.17 |  | | | 3.22 | | 0.16 | |  | | Mean difference: 0.40, 95% CI [-0.07, 0.86], *Wald* = 1.67, *df* = 85, *p* = .07, *d* = 0.39 |  |
| *Original Scale* | 8.88 |  |  | 8.73 |  |  |  |  | 12.04 | |  |  | | | 9.34 | |  | |  | |  |  |
|  |  |  |  |  |  |  |  |  |  | |  |  | | |  | |  | |  | |  |  |
| Non-Specific AEs | 2.32 | 0.15 |  | 2.32 | 0.15 |  |  | | 2.62 | | 0.15 |  | | | 2.17 | | 0.15 | |  | |  |  |
| *Original scale* | 4.40 |  |  | 3.04 |  |  |  |  | 5.86 | |  |  | | | 3.69 | |  | |  | |  |  |
|  |  |  |  |  |  |  |  |  |  | |  |  | | |  | |  | |  | |  |  |
| Global AE scale | 4.55 | 0.45 | 35 | 4.03 | 0.43 | 40 |  | | 5.35 | | 0.45 | 35 | | | 4.33 | | 0.43 | | 40 | |  |  |
| Correct description of the nocebo effect | | |  |  |  |  |  | |  | |  |  | | |  | |  | |  | |  |  |
| AEs | 14.93 | 1.16 | 51 | 13.37 | 1.21 | 46 |  | | 19.47 | | 1.16 | 51 | | | 14.94 | | 1.22 | | 46 | |  |  |
| Specific AEs | 3.14 | 0.13 |  | 3.08 | 0.14 |  |  | | 3.63 | | 0.13 |  | | | 3.23 | | 0.14 | |  | |  |  |
| *Original Scale* | 8.89 |  |  | 8.51 |  |  |  |  | 12.17 | |  |  | | | 9.44 | |  | |  | |  |  |
|  |  |  |  |  |  |  |  |  |  | |  |  | | |  | |  | |  | |  |  |
| Non-Specific AEs | 2.30 | 0.13 |  | 2.30 | 0.13 |  |  | | 2.61 | | 0.13 |  | | | 2.19 | | 0.13 | |  | |  |  |
| *Original scale* | 4.27 |  |  | 3.08 |  |  |  |  | 5.82 | |  |  | | | 3.81 | |  | |  | |  |  |
|  |  |  |  |  |  |  |  |  |  | |  |  | | |  | |  | |  | |  |  |
| Global AE scale | 4.44 | 0.37 | 51 | 3.83 | 0.38 | 46 |  | | 5.35 | | 0.37 | 51 | | | 4.33 | | 0.41 | | 46 | |  |  |
| Patients without cancer progression | | |  |  |  |  |  | |  | |  |  | | |  | |  | |  | |  |  |
| AEs | 15.54 | 1.19 | 47 | 13.44 | 1.16 | 48 |  | | 19.79 | | 1.19 | 47 | | | 14.82 | | 1.18 | | 48 | |  |  |
| Specific AEs | 3.20 | 0.14 |  | 3.09 | 0.14 |  |  | | 3.65 | | 0.14 |  | | | 3.23 | | 0.14 | |  | |  |  |
| *Original Scale* | 9.21 |  |  | 8.52 |  |  |  |  | 12.35 | |  |  | | | 9.40 | |  | |  | |  |  |
|  |  |  |  |  |  |  |  |  |  | |  |  | | |  | |  | |  | |  |  |
| Non-Specific AEs | 2.39 | 0.13 |  | 2.39 | 0.13 |  |  | | 2.64 | | 0.13 |  | | | 2.18 | | 0.13 | |  | |  |  |
| *Original scale* | 4.73 |  |  | 3.17 |  |  |  |  | 5.97 | |  |  | | | 3.76 | |  | |  | |  |  |
|  |  |  |  |  |  |  |  |  |  | |  |  | | |  | |  | |  | |  |  |
| Global AE scale | 4.50 | 0.39 | 47 | 3.88 | 0.38 | 48 |  | | 5.51 | | 0.39 | 47 | | | 4.25 | | 0.40 | | 48 | |  |  |

*Note.* Pooled means and standard errors of linear mixed models after adjustment for distress and cancer staging. The primary outcome adverse effects were computed as a sum-score of 7 symptoms x 10-point severity-scale resulting in a scale range of 0 to 70. Accordingly, its (non-transformed) subscales specific AE and non-Specific AE have ranges of 0-40 and 0-30, respectively. The global scale ranged from 0 to 10. Means on the original scales were obtained by back transforming the estimates. Significant differences were indicated in bold.

AE = adverse effect; T2 = 10 days after onset of chemotherapy; T3 = 12 weeks after onset of chemotherapy; CG = attention control group; EG = nocebo education group; CI = confidence interval, d = Cohen’s d.
